# Temperature Dependence of *Plasmodium falciparum* Erythrocytic Stage Development

**DOI:** 10.4269/ajtmh.18-0894

**Published:** 2019-04-01

**Authors:** Yutatirat Singhaboot, Srisuda Keayarsa, Nattaporn Piaraksa, Weerapong Phumratanaprapin, Parinya Kunawut, Arjen Dondorp, Kesinee Chotivanich

**Affiliations:** 1 Department of Clinical Tropical Medicine, Faculty of Tropical Medicine, Mahidol University, Bangkok, Thailand;; 2 Department of Medicine, Faculty of Medicine Ramathibodi Hospital, Mahidol University, Bangkok, Thailand;; 3 Mahidol Oxford Tropical Medicine Research Unit, Faculty of Tropical Medicine, Mahidol University, Bangkok, Thailand;; 4 Centre for Tropical Medicine, Churchill Hospital, Oxford, United Kingdom

## Abstract

*Plasmodium falciparum* infection causes febrile illness and severe disease with multiple organ failure and death when treatment is delayed. Antipyretic treatment is standard, and inducing hypothermia has been proposed to protect the brain in cerebral malaria. Here, we investigated the temperature dependence of asexual-stage parasite development and parasite multiplication in vitro. *Plasmodium falciparum* laboratory strain TM267 was incubated for 2 hours (short exposure) or 48 hours (continuous exposure) at different temperatures (32°C, 34°C, 35°C, 38°C, 39°C, and 40°C). The starting parasite developmental stage (ring, trophozoite, or schizont) varied between experiments. The parasite multiplication rate (PMR) was reduced under both hyper- and hypothermic conditions; after continuous exposure, the mean PMR ± SD was 9.1 ± 1.2 at 37°C compared with 2.4 ± 1.8 at 32°C, 2.3 ± 0.4 at 34°C, and 0.4 ± 0.1 at 40°C (*P* < 0.01). Changes in PMR were not significant after 2-hour exposure at temperatures ranging from 32°C to 40°C. Morphological changes in parasite cytoplasm and nucleus could be observed after long exposure to low or high temperature. After 48-hour incubation, rosette formation (≥ 2 uninfected red blood cells bound to infected red blood cells) was decreased at 34°C or 39°C compared with that at 37°C. In conclusion, both hyper- and hypothermia reduce PMR and delay erythrocytic stage development of *P. falciparum*, subsequently reducing rosette formation.

## INTRODUCTION

*Plasmodium falciparum* malaria remains a leading cause of death in the tropical world. Among all human malaria species, most cases of severe malaria with multiple organ failure are caused by this parasite.^1–4^ Fever is the key symptom; the classic description of a regular tertian pattern is observed in 25% of cases. Compared with adult patients, children are more prone to high fever (> 40°C), that is, often accompanied by febrile convulsions. Fever also contributes to nausea and vomiting, which may compromise treatment with oral antimalarial drugs. Because of this, antipyretic therapy with paracetamol or tepid sponging is recommended. However, it has been argued that antipyretic therapy with paracetamol prolongs the parasite clearance time after antimalarial treatment, although this was not confirmed in a more recent study.^5,6^ To assess the benefit of antipyretic therapy, it is important to determine whether temperature affects the growth and multiplication of asexual-stage *P. falciparum* parasites because the total body parasite biomass is one of the main determinants of disease severity.^7,8^ In vivo and in vitro studies suggest that parasites obtained from patients with severe disease have a higher parasite multiplication rate (PMR),^9–12^ and *P. falciparum* isolates from patients with severe malaria show higher in vitro PMRs than those with uncomplicated malaria.^12^ Previous studies have shown that hypothermic conditions (28–32°C) delayed the erythrocytic life cycle development of *P. falciparum*,^13,14^ whereas febrile temperature (40°C) inhibited parasite growth^15,16^ in vitro. In addition, it is important to assess the effect of temperature on parasitized red blood cell (PRBC) adhesion properties affecting microcirculatory blood flow. Microcirculatory impairment is central in the pathogenesis of severe falciparum malaria and is caused by cytoadherence of PRBCs to vascular endothelium (causing sequestration) and to uninfected red blood cells (RBCs) (causing rosette formation).^17–19^ Furthermore, it has been shown that fever causes PRBCs to cytoadhere earlier in their 48-hour asexual life cycle.^20^ In this study, we conducted a comprehensive systematic investigation of the impact of hyper- and hypothermic culture conditions on *P. falciparum* growth and rosette formation.

## MATERIALS AND METHODS

### Parasite culture.

*Plasmodium falciparum* laboratory strain TM267 was cultured under standard conditions,^21^ and parasites were synchronized to the ring stage by treatment with 5% D-sorbitol. Red blood cell suspensions containing 1% parasitemia at 3% hematocrit were cultured in a candle jar and then incubated under various temperatures. Incubators were set up to simulate hypothermic conditions (32°C, 34°C, and 35°C) and hyperthermic conditions (38°C, 39°C, and 40°C). The temperature variation was ±0.5°C. The temperature at 37°C was set as the control, and the culture medium was changed daily. In these experiments, the incubation temperature was changed to hypo- or hyperthermic conditions, either for the duration of the full 48-hour experiment (continuous exposure) or for 2 hours followed by continued incubation for 48-hours at standard conditions at 37°C (short exposure). Parasite growth was examined by counting the number of parasites per 5,000 RBCs on thin blood smears using Field’s stain by light microscopy at a magnification of ×100 using oil immersion. *Plasmodium falciparum* parasites were assessed for developmental stages that divide the developmental cycle of the parasite into eight stages (tiny, small, and large rings; early, mid, and late trophozoites; and early and late schizonts) based on cytoplasm morphology, appearance of malaria pigment, and number of nuclei as described previously.^22^ Each experiment was performed in triplicate; results are expressed as mean ± SD.

### Erythrocyte preparation.

Healthy donors provided 5 mL of whole blood collected in citrate phosphate dextrose tubes. Packed RBCs were obtained by centrifugation at 2,500 rpm for 5 minutes and removal of plasma and buffy coat. The packed RBCs were then resuspended in malaria complete medium and stored at 4°C until further use. PMR was calculated using the following formula: PMR = % parasitemia after schizogony at 48 hours divided by % starting parasitemia.

### Rosette formation.

Rosette formation was assessed in RBC suspensions containing trophozoite-infected RBCs; 15 µL of RBC suspension was dropped onto a microscope slide, covered by a glass slip, and rosette formation was quantified using light microscopy. Rosette formation or adhesion of ≥ 2 uninfected RBCs to a parasite-infected RBC was quantified as described previously.^23,24^ The numbers of rosettes were counted per 100 infected RBCs under light microscopy at high magnification (×1,000).

### Statistical analysis.

Differences between parasite growth at low and high temperatures compared with that at standard temperature (37°C) was assessed by the paired-sample *t*-test. Non-normally distributed data were analyzed by the Mann–Whitney U test. *P*-values < 0.05 were considered statistically significant.

## RESULTS

### Continuous exposure to hyper- and hypothermic conditions.

**Figure 1.**
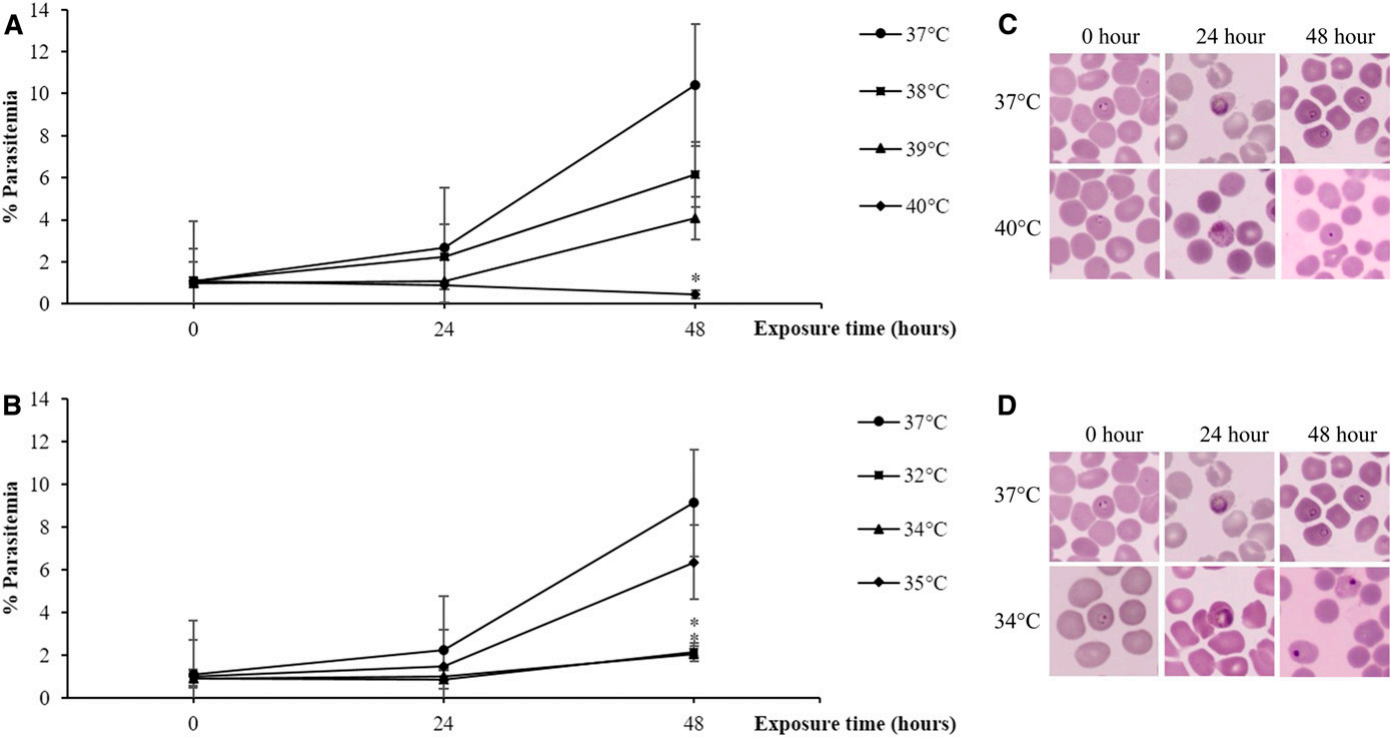
Comparison of parasite growth between in vitro cultures of *Plasmodium falciparum* laboratory strain TM267 grown under different continuous hyper- and hypothermic conditions for 48 hours (*N* = 3). Data are presented as % parasitemia (number of infected red blood cells per 5,000 red blood cells). (**A**) Under hyperthermic conditions, % parasitemia was significantly decreased at 40°C but was not significantly different at 38°C and 39°C. (**B**) Under hypothermic conditions, parasitemia was reduced at 32°C and 34°C but not at 35°C. *Plasmodium falciparum* morphology presented as condensed, pyknotic nuclei after continuous exposure at 40°C (**C**) and 34°C (**D**). Thin blood smears stained using Field’s stain were visualized under light microscopy at ×1,000 magnification. *P < 0.05. This figure appears in color at www.ajtmh.org.

In the continuous 48-hour exposure experiment, the starting parasitemia was 1% (range 0.9–1.1%). For culture under continuous hyperthermic and hypothermic conditions, parasite maturation assessed after 48 hours was not significantly changed when compared with the standard culture at 37°C (*P* = 0.06). PRM was calculated by comparing parasitemia at 48 hours with baseline parasitemia (Figure 1A: hyperthermia, Figure 1B: hypothermia). The standard mean PMR ± SD at 37°C was 9.1 ± 1.2. The mean ± SD PMR at 40°C was significantly decreased to 0.43 ± 0.1 (*P* = 0.04); however, PMRs were not significantly different at 38°C and 39°C (5.7 ± 1.2, *P* = 0.05 and 4.1 ± 1.6, *P* = 0.10, respectively). Under hypothermic conditions, the mean ± SD PMR was reduced at 32°C to 2.4 ± 1.8 (*P* = 0.01) and at 34°C to 2.3 ± 0.4 (*P* = 0.05), but not at 35°C (6.4 ± 2.0, *P* = 0.37). Parasites with condensed, pyknotic-appearing nuclei were observed at hyper- and hypothermic conditions after 48-hour exposure (Figure 1C and D).

### Short exposure to hyper- and hypothermic conditions.

**Figure 2.**
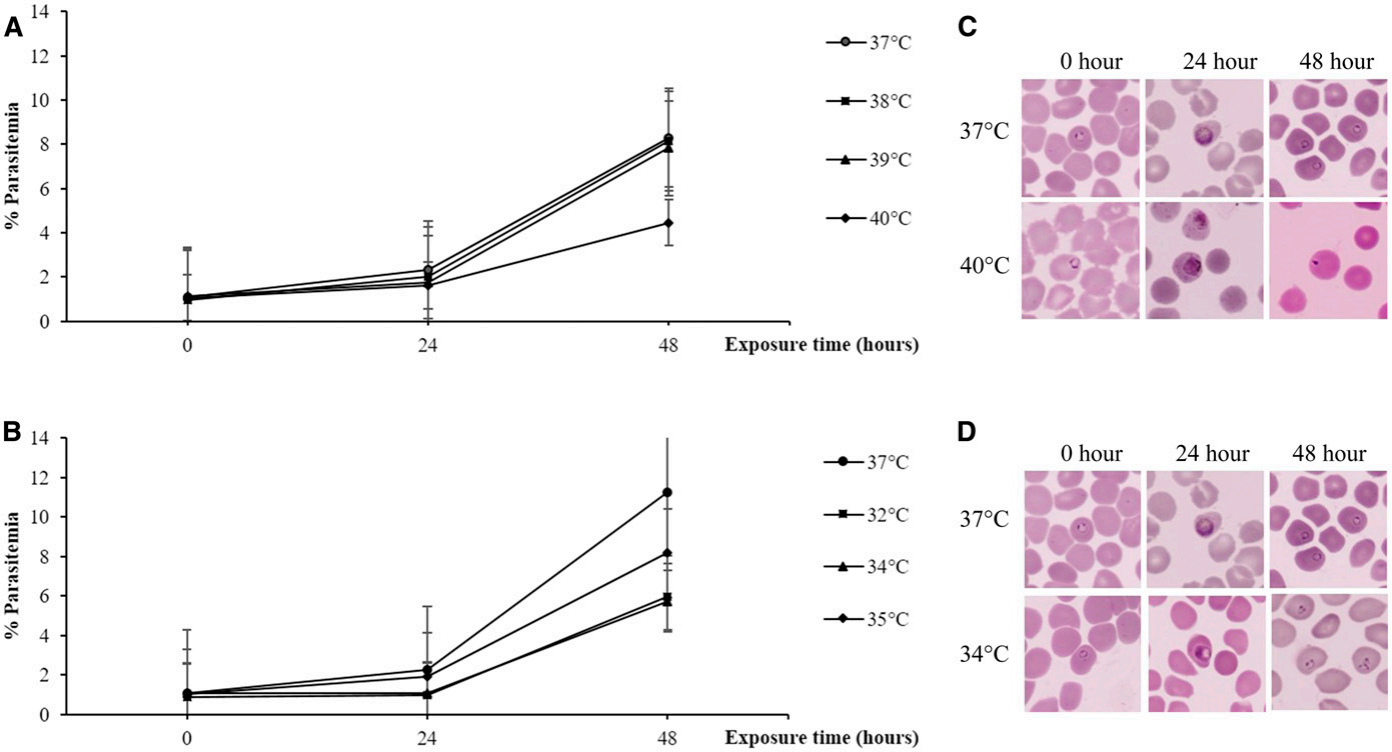
Comparison of parasite growth between in vitro cultures of *Plasmodium falciparum* laboratory strain TM267 grown under different hyper- and hypothermic conditions for 2 hours, followed by continuous culture at 37°C for 48 hours. Data are presented as % parasitemia (number of infected red blood cells per 5,000 red blood cells). (**A**) Under hyperthermic and (**B**) hypothermic conditions, the % parasitemia at hyper- and hypothermic conditions was not significantly changed. *Plasmodium falciparum* morphology presented as condensed, pyknotic nuclei under hyperthermic conditions (**C**) and normal morphology under hypothermic conditions (**D**). Thin blood smears stained using Field’s stain were visualized under light microscopy at ×1,000 magnification. This figure appears in color at www.ajtmh.org.

In short exposure experiments, the starting parasitemia was 1% (range 0.9–1.1%). PMR was calculated by comparing parasitemia at 48 hours compared with baseline parasitemia (Figure 2A: hyperthermia, Figure 2B: hypothermia). After 2-hour culture under hyperthermic and hypothermic conditions followed by continuing culture at 37°C, parasite maturation assessed at 48 hours was similar to the standard culture at 37°C. The mean PMR ± SD of control (37°C) was 8.8 ± 2.1, at 38°C was 8.4 ± 0.3 (*P* = 0.67), at 39°C was 7.0 ± 0.2 (*P* = 0.94), and at 40°C was 4.1 ± 0.6 (*P* = 0.07). At hypothermia, the mean PMR ± SD at 32°C was 6.6 ± 2.7 (*P* = 0.86), at 34°C was 5.3 ± 2.7 (*P* = 0.50), and at 35°C was 8.2 ± 3.2 (*P* = 0.58). Pyknotic nuclei could be observed under hyperthermic (40°C) culture conditions but not under hypothermic culture condition (Figure 2C and D).

### Rosette formation under hyper- or hypothermic conditions.

**Figure 3.**
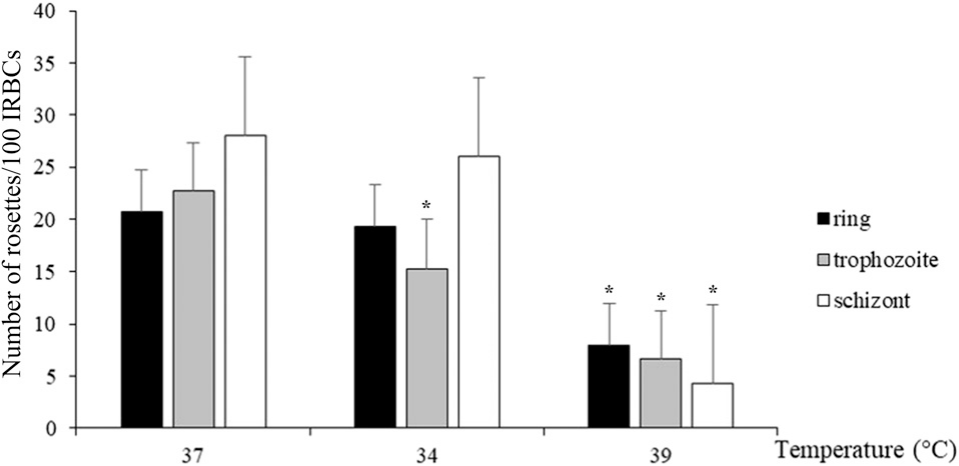
*Plasmodium falciparum* rosette formation under hyper- and hypothermic conditions. Data are presented as number of rosettes per 100 infected red blood cells (IRBCs). Parasite culture starting at ring and schizont stages showed significantly reduced rosette formation at 40°C but remained unchanged under hypothermic conditions. Parasite culture starting at the trophozoite stage showed significantly decreased rosette formation under hyper- and hypothermic conditions. *P < 0.05.

Rosette formation assessed at the moment parasites had developed from the initial small ring stage to trophozoite stage after 24 hours showed a decrease in the number of rosettes formed under hyperthermic conditions (Figure 3). The mean ± SD number of rosettes per 100 infected RBCs at 37°C was 23 ± 2, whereas at 39°C this was reduced to 8 ± 7 (*P* = 0.01). There was no difference under hypothermic conditions (34°C), with a mean ± SD number of rosettes formed of 19 ± 3 (*P* = 0.40). Rosettes in parasite culture starting with trophozoite stage and assessed 48 hours later showed a decrease in rosette formation under both hyper- and hypothermic conditions. The mean ± SD number of rosettes formed at 37°C was 23 ± 2, whereas at 34°C, this was 15 ± 1 (*P* = 0.03) and at 39°C, this was 7 ± 3 (*P* = 0.01). In parasite culture starting with immature schizont-stage parasites (containing 3–5 merozoites per schizont), rosettes assessed 36 hours after schizogony in the next erythrocytic cycle (trophozoite stage) showed a decrease in rosette formation under hyperthermic, but not hypothermic, conditions. The mean ± SD number of rosettes at 37°C was 28 ± 2 compared with 26 ± 2.2 at 34°C (*P* = 0.45) and 4 ± 4 at 39°C (*P* = 0.01).

## DISCUSSION

Blockage of the microcirculation by sequestered PRBCs is the central cause of organ failure in severe falciparum malaria. Other systemic manifestations, such as fever, are attributed to pro-inflammatory cytokines released in response to the parasite, plasmodial DNA, and red cell membrane products.^25^ Plasmodial DNA is presented through hemozoin produced by the parasite, which interacts with Toll-like receptor 9, leading to the release of pro-inflammatory cytokines that in turn induce cyclooxygenase-2-upregulated prostaglandins, subsequently causing fever.^26,27^ It should be noted that the pro-inflammatory cytokine response does not only cause fever, but can also contribute to endothelial changes including increased expression of receptors for PRBC adhesion^28^ and host cell apoptosis.^29^ In the present study, we show that continuous exposure of *P. falciparum* in an in vitro culture under hyperthermic conditions reduces the PMR and changes parasite morphology. High fever might contribute to parasite killing during falciparum malaria infection. A previous study has shown inhibition of in vitro growth of *P. falciparum* at 40°C, and our results support this finding.^15^ Short exposure to hyperthermia, mimicking a fever spike, did not significantly decrease the PMR in vitro. It had been reported that *P. falciparum* was least affected when incubated at high temperature for a short period (1–6 hours).^16,30^ This suggests that *P. falciparum* can resist short-term, but not long-term, hyperthermia associated with malarial infection. A possible mechanism is the effective heat shock protein (Hsp) response in *P. falciparum*. Heat shock protein is a 90-kDa protein complex (PfHsp90), which consists of PfHsp70, PfPP5, tubulin, and other proteins.^31^ PfHsp90 functions as an adenosine triphosphate-dependent molecular chaperone that is responsible for stabilizing misfolded proteins during heat stress. In this way, PfHsp90 protects the parasite when stressed by elevated temperatures.^32^

Fever is an inherent symptom of malaria and contributes to general unwell-being, nausea, vomiting, and, in small children, febrile convulsions. Paracetamol (acetaminophen) is the recommended antipyretic therapy as it is safe, inexpensive, and widely available.^33^ However, paracetamol has been associated with a prolonged parasite clearance time, which our study suggests could be, to some extent, explained by increased survival in normothermia.^5^ A more recent randomized controlled trial did not show a difference in parasite clearance between patients treated with or without paracetamol.^6^ Our study also showed that culture under continuous hypothermic conditions reduced parasite growth rate and induced morphological changes, including pyknotic nuclei. This effect was not observed after short exposure to hypothermia. A previous study has also shown that mild hypothermia (32°C) inhibits in vitro growth of *P. falciparum*.^14^ A similar finding was reported, where *P. falciparum* was cultured at 28°C for 66 hours, delaying parasite development, with restoration of growth properties during follow-up culture at 37°C.^13^ Mild hypothermia has been suggested as an adjunctive brain protective treatment of cerebral malaria, which could also contribute to parasite killing. In the present study, rosette formation under hyper- and hypothermic conditions was decreased. Rosette formation, which produces clusters of infected erythrocytes with uninfected ones, has been associated with increased parasite multiplication by supporting parasite invasion. In particular, rosette formation can shield parasitized erythrocytes, subsequently supporting immune evasion, and may contribute to microcirculatory flow impairment.^34^ Fever might, thus, attenuate these harmful effects through reducing rosette formation.

In conclusion, prolonged hyper- and hypothermic in vitro growth conditions reduced the PMR and delayed the erythrocytic stage development of a laboratory strain of *P. falciparum*. Parasite cytoplasm distortion and pyknotic nuclei were observed after long exposures to low and high temperatures. Furthermore, hyperthermia reduced rosette formation (≥ 2 uninfected RBCs bound to infected RBCs). Fever might, thus, be an adaptive host response to *P. falciparum* infection.
